# Neuron Glia-Related Cell Adhesion Molecule (NrCAM) Promotes Topographic Retinocollicular Mapping

**DOI:** 10.1371/journal.pone.0073000

**Published:** 2013-09-02

**Authors:** Jinxia Dai, Mona Buhusi, Galina P. Demyanenko, Leann H. Brennaman, Martin Hruska, Matthew B. Dalva, Patricia F. Maness

**Affiliations:** 1 Department of Biochemistry and Biophysics, University of North Carolina School of Medicine, Chapel Hill, North Carolina, United States of America; 2 Thomas Jefferson University, Department of Neuroscience, Jefferson Hospital for Neuroscience, Philadelphia, Pennsylvania, United States of America; Institut de la Vision, France

## Abstract

NrCAM (Neuron-glial related cell adhesion molecule), a member of the L1 family of cell adhesion molecules, reversibly binds ankyrin and regulates axon growth, but it has not been studied for a role in retinotopic mapping. During development of retino-collicular topography, NrCAM was expressed uniformly in retinal ganglion cells (RGCs) along both mediolateral and anteroposterior retinal axes, and was localized on RGC axons within the optic tract and superior colliculus (SC). Anterograde tracing of RGC axons in NrCAM null mutant mice at P10, when the map resembles its mature form, revealed laterally displaced ectopic termination zones (eTZs) of axons from the temporal retina, indicating defective mediolateral topography, which is governed by ephrinB/EphBs. Axon tracing at P2 revealed that interstitial branch orientation of ventral-temporal RGC axons in NrCAM null mice was compromised in the medial direction, likely accounting for displacement of eTZs. A similar retinocollicular targeting defect in EphB mutant mice suggested that NrCAM and EphB interact to regulate mediolateral retino-collicular targeting. We found that EphB2 tyrosine kinase but not an EphB2 kinase dead mutant, phosphorylated NrCAM at a conserved tyrosine residue in the FIGQY ankyrin binding motif, perturbing ankyrin recruitment in NrCAM transfected HEK293 cells. Furthermore, the phosphorylation of NrCAM at FIGQY in SC was decreased in EphB1/3 and EphB1/2/3 null mice compared to WT, while phospho-FIGQY of NrCAM in SC was increased in EphB2 constitutively active (F620D/F620D) mice. These results demonstrate that NrCAM contributes to mediolateral retinocollicular axon targeting by regulating RGC branch orientation through a likely mechanism in which ephrinB/EphB phosphorylates NrCAM to modulate linkage to the actin cytoskeleton.

## Introduction

Topographic targeting of retinal ganglion cells (RGCs) to the superior colliculus (SC) of the midbrain is a developmental process in which precise spatial ordering of RGC axon terminations is established along orthogonal axes. RGC axons from temporal-nasal axis of the retina target along the anterior-posterior SC axis, while dorsal-ventral RGC axons target along lateral-medial SC axis. Studies on the mechanism by which this highly ordered mapping is achieved have indicated a vital function of ephrin/Eph signaling in axon guidance. Complementary gradients of ephrinA ligands in the SC and EphA receptors in the retina control the mapping of temporal-nasal RGC axons along anterior-posterior axis of the SC, while counter gradients of ephrinBs in the SC and EphBs in the retina play a major role in dorsal-ventral mapping of RGC axons along the mediolateral axis of the SC [1]–[3]. Repulsive Wnt/Ryk signaling contributes to mediolateral mapping by counterbalancing ephrinB/EphB-mediated medial attraction [4]. However, these molecular determinants do not fully account for retinotopic mapping.

NrCAM is a member of the L1 family of immunoglobulin-class cell adhesion molecules (L1, NrCAM, CHL1, Neurofascin), and has important functions in axon guidance and myelination during brain development [5]. NrCAM is implicated in neuropsychiatric disorders including autism [6]–[10] and schizophrenia [11], [12], as well as addiction-related behaviors [13], [14]. NrCAM mediates topographic targeting of thalamocortical axons [15], and ipsilateral guidance of RGC axons at the optic chiasm [16] but a role in retino-collicular mapping has not been examined. The closely related molecule L1 is required for topographic targeting of RGC axons along both mediolateral and anteroposterior axes of the SC [17], [18]. NrCAM and L1 associate with ALCAM, an immunoglobulin-class adhesion molecule that is expressed as a substrate-bound ligand in the SC and functions in mediolateral retinocollicular mapping [19]. The intracellular region of NrCAM harbors a motif (FIGQY) that is highly conserved among L1 family members, and reversibly engages the actin adaptor ankyrin [20]. L1 mutant mice with a pathogenic substitution of tyrosine^1229^ for histidine in the FIGQY^1229^ motif exhibit mediolateral retinocollicular mapping defects due to loss of ankyrin binding [18]. Similar mediolateral targeting defects of retinocollicular axons in EphB receptor mutant mice [21], [22] suggest a possible combinatorial interaction among L1 family members and ephrinB/EphBs in development of retinotopy.

To determine if NrCAM contributes to retino-collicular topography, we analyzed NrCAM null mutant mice [23] by axon tracing during the postnatal development of the retinotopic map. We found that loss of NrCAM resulted in mistargeting of temporal RGC axons along the mediolateral SC axis, and compromised the ability of RGC axons to medially orient their interstitial branches. Moreover, EphB receptors were found to induce phosphorylation of NrCAM on the tyrosine residue within the FIGQY ankyrin binding motif, inhibiting ankyrin recruitment. Furthermore, NrCAM phospho-FIGQY levels in the SC were decreased in EphB1/3 and EphB1/2/3 null mice and increased in mutant mice overexpressing constitutively active EphB2 kinase. These results are consistent with the interpretation that ephrinB/EphB signaling regulates NrCAM-ankyrin binding to modulate interstitial branch attraction within the SC necessary for proper mapping of RGC axon subpopulations along the mediolateral SC axis.

## Materials and Methods

### Ethics Statement

Mice were maintained and handled ethically according to all of the Institutional Animal Care and Use Committee policies of the University of North Carolina at Chapel Hill (PHS Animal Welfare Assurance number, A3410-01). The UNC IACUC specifically approved this study (protocol no.10.030).

### Mice

NrCAM null mutant (knockout) mice were originally generated in the W4 ES cell line 129S6/EvSvTaconic by homologous recombination, and maintained on a hybrid background of 129S6/EvSvTaconic and Swiss Webster (CFW), as reported previously [23]. NrCAM null (KO) and wild-type (WT) littermates were generated by intercrossing NrCAM heterozygotes and genotyping offspring by PCR.

### Axon Tracing and Analysis

Anterograde tracing of retinocollicular axon was performed as described [17]–[19,21]. Briefly, NrCAM null mutants and WT littermates at P2 or P8 were focally injected with a DiI tracer (10% in dimethylformamide) into the peripheral retina by a picospritzer. After 24 hour (labeled at P2) or 48 hour (labeled at P8), mice were anesthetized and perfused transcardially with 4% paraformaldehyde (PFA) in 0.1 M phosphate-buffered saline (PBS). The injection site was identified according to the insertion of the extraocular muscles (lateral and inferior recti). Afterwards, the retina was dissected out and demarcated the quadrants. To verify the injection sites and analyze the termination zones (TZs), the injected retina, superior and inferior colliculi were whole mounted onto slides and observed by epifluorescence and confocal microscopy. Those with injection sites covering about 3–5% of the retina were analyzed. Termination zones of labeled RGC axons in the SC were identified by their densely branched appearance.

To quantitatively analyze branch orientation of VT RGC axons at P3, the SC was divided into three bins: lateral to TZ, within TZ and medial to TZ as described previously [17]–[19], [21]. Only mutants with one normally positioned TZ were analyzed. The total number of labeled axons and branches were counted in confocal microscopy, and medial or lateral branch orientation for each bin was recorded. For each bin, the number of medially oriented branches minus that of laterally oriented branches was divided by the total number of branches to reach the final directional coefficient (DC). The DC for each bin was compared between WT and NrCAM null mice by single-factor ANOVA with significance at p<0.05.

To analyze the entrance location of VT RGC axons in SC, the SC of WT and NrCAM null mice was divided into ten bins: four bins medial to the TZ and six bins lateral to the TZ as described previously [17]–[19], [21]. For each bin, the percent of axons entering the SC was scored for each mouse and averaged over groups. The entrance location of RGC axons was compared between WT and NrCAM null mice by single-factor ANOVA (p<0.05).

### Cell Culture, Immunoprecipitation and Immunoblotting

HEK293T cells were cultured in DMEM with 10% bovine fetal serum (FBS). Plasmids encoding mouse NrCAM, chicken EphB2 and EphB2 kinase dead mutant (EphB2K662R; EphB2 KD) [24], mouse EphB1with a hemagglutinin epitope (HA) tag, mouse EphB2, and mouse EphB3 with HA tag were transfected into HEK293T cells using Lipofectamine 2000 (Invitrogen). After 48 hours, cells were washed with Hank’s Balanced Salt Solution and lysed in RIPA buffer containing protease inhibitors and phosphatase inhibitors (1% NP-40, 1% sodium deoxcholate, 0.1% sodium dodecyl sulfate, 0.15 M NaCl, 5 mM Na-EDTA, 1 mM Na-EGTA in 20 mM Tris-HCl, pH 7.0, 10 µg/mL leupeptin, 0.11 TIU/mL aprotinin, 0.2 mM sodium orthovanadate, and 10 mM sodium fluoride**).** The SC (P2-P3) was dissected from WT, EphB1/3 double null mice, EphB1/2/3 triple null mice [25], [26], and from homozygous mutant mice expressing a constitutively overactive EphB2 kinase (F620D) that harbors a point mutation (F620D) conferring forward signaling independent of ligand binding [27], [28]. SC preparations were homogenized in RIPA buffer containing protease and phosphatase inhibitors.

Lysates (500 ug) were immunoprecipitated with rabbit anti-NrCAM antibody (5 ug; Abcam, ab24344) or normal rabbit IgG for 2 hour at 4°C, then incubated with protein A/G-Sepharose beads (Pierce Biotechnology, Rockford, IL) for 1 hour. Beads were washed with RIPA buffer, and proteins were eluted by boiling in SDS-PAGE sample buffer. The immunoprecipitates were resolved on 7.5% SDS-PAGE gels and transferred to nitrocellulose membranes. After blocking in 5% non-fat dry milk, the membranes were immunoblotted with rabbit anti-p-FIGQY antibody (1∶500), and reprobed with rabbit anti-NrCAM (0.4 ug/ml; Abcam, ab24344), mouse anti-EphB2 (1∶250; Invitrogen, 37–1700) or mouse anti-HA (1∶1000; BaBco, HA-11) antibodies. Bands were developed on X-ray film by enhanced chemiluminescence (Perkin-Elmer, Waltham, MA).

### Immunohistochemistry

Postnatal WT mice at day 0 (P0) and day 6 (P6) were anesthetized by hypothermia and perfused transcardially with 4% PFA/PBS. The brains or entire heads were post-fixed in 4% PFA in PBS overnight at 4°C and sectioned in a cryostat after cryoprotection with 30% sucrose in PBS. After blocking in 2% BSA/10% normal donkey serum/0.2% Triton x-100 in PBS at room temperature for 2 hours, sections were incubated with rabbit anti-NrCAM polyclonal antibody (1∶200; Abcam, AB24344), rat anti-L1 monoclonal antibody (1∶200; Millipore, MAB5272), or mouse anti-neurofilament 165 antibodies (1∶500; Developmental Studies Hybridoma Bank, 2H3), or a combination of rat anti-L1 and mouse anti-neurofilament 165 at room temperature for 2 hours. Signals were developed by incubating sections with FITC-conjugated donkey anti-rat or anti-rabbit IgG (1∶200; Jackson Immunoresearch) with Cy3-conjugated donkey anti-rabbit or anti-mouse IgG (1∶200; Jackson Immunoresearch) for 1 hour at room temperature. Images were obtained by Zeiss 710 confocal microscopy after DAPI counterstaining.

### NrCAM-ankyrin Recruitment Assay

Ankyrin recruitment to NrCAM in the plasma membrane was performed as described previously for L1-ankyrin recruitment [29], [30]. HEK293T cells were cultured on poly-D-lysine coated Mat-Tek dishes in DMEM-10% FBS and transfected with plasmid pEGFP-N1 expressing ankyrinG-EGFP fusion protein [31], pcDNA3 plasmid expressing rat NrCAM, and/or pcDNA3 plasmid expressing chicken EphB2 or the EphB2 kinase dead mutant [24] using lipofectamine 2000 (Invitrogen). Expression of EphB receptors in HEK293T cells results in autoactivation, typical for receptor tyrosine kinases, such that treatment with ephrinB1 ligands does not further increase phospho-FIGQY levels [32]. After 18 hours, cells were fixed in 4% PFA/PBS and blocked in 10% normal donkey serum+2%BSA+0.05% Triton X-100 in PBS. Cells were subjected to immunofluorescence staining with antibodies against the extracellular region of NrCAM (Abcam, ab24344; 1∶400) or EphB2 (Invitrogen, 36–6100; 1∶200) using Cy3 donkey anti-rabbit secondary antibody. Confocal images were captured using the 488 nm and 543 nm excitation lines of the lasers for ankyrin-EGFP and Cy3, respectively. Cells were scored in 3 replicate cultures for the percent of cells that display ankyrin-EGFP recruited to the cell surface using criteria described in [29]. Means ± S.E.M. were compared for statistically significant differences using one-way ANOVA and Tukey’s post-hoc comparisons (p<0.001).

## Results

### NrCAM is Expressed in Retinal Axon Subpopulations during Retinocollicular Mapping

**Figure 1:**
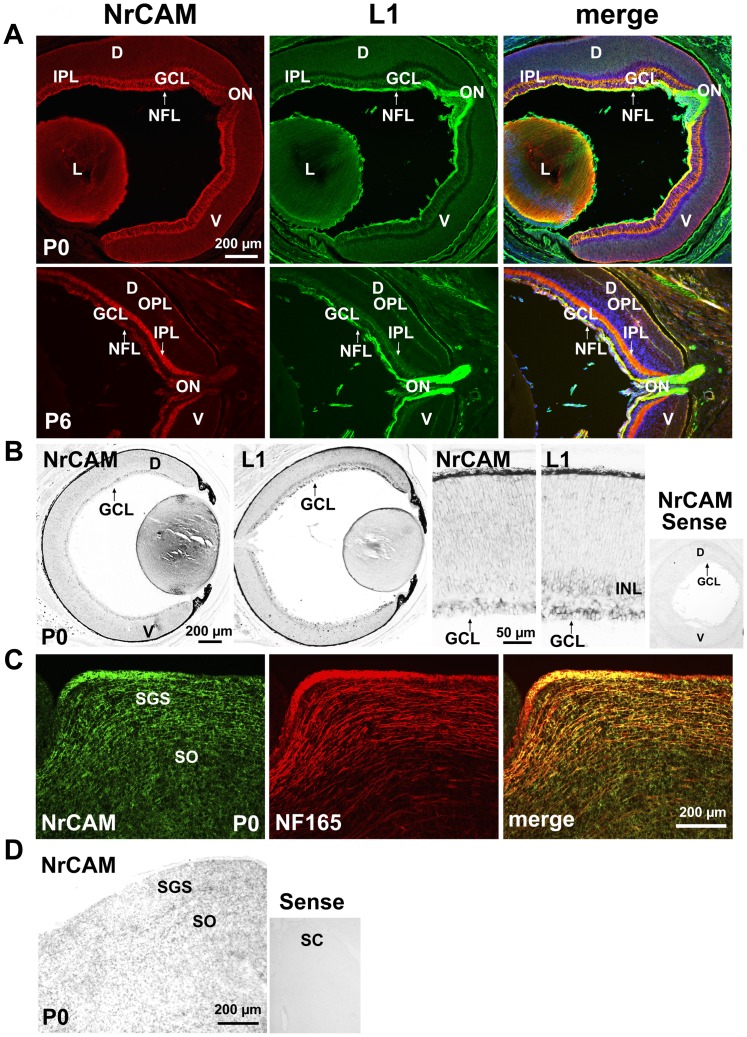
Early Postnatal Expression of NrCAM in the Retina and Superior Colliculus. **A.** Immunofluorescence staining for NrCAM (red) in mouse retina at P0 and P6 demonstrated NrCAM localization in fibers of the retinal NFL, ON, IPL, and lens, as shown in confocal images. Double staining for L1 (green) showed partial co-localization with L1. GCL, ganglion cell layer; NFL, nerve fiber layer; IPL, inner plexiform layer; ON, optic nerve head; OPL, outer plexiform layer; L, lens; D, dorsal; V, ventral. **B.** *In situ* hybridization with antisense probes in the mouse retina (P0) showed NrCAM transcripts in cell bodies in the GCL. L1 transcripts were present in the GCL and inner nuclear layer (INL). Background labeling of retina is shown by hybridization with the NrCAM sense probe. **C.** Immunofluorescence staining for NrCAM in sagittal sections of SC at P0 showed partial co-localization with the axonal marker NF165 in a portion of RGC axons in the SGS and SO, as well as some additional labeling within the SC. SGS, stratum griseum superficiale; SO, stratum opticum. **D.** *In situ* hybridization with antisense probe showed NrCAM transcripts in cell bodies located within the SC (P0, sagittal sections). Background is indicated by hybridization with the NrCAM sense probe.

Retinocollicular mapping in the mouse begins around embryonic day 14 (E14), when RGC axons first reach the anterior SC. Axons subsequently grow into the stratum griseum superficiale (SGS) and stratum opticum (SO) layers of the SC by postnatal day 0 (P0), overshooting their final termination zones (TZs) [33], [34]. RGC axons remodel postnatally from P0 to P10 by a combination of interstitial branching and axon retraction to form correctly targeted final TZs in the SC. To determine if NrCAM might play a role in RGC axon mapping, NrCAM expression was analyzed during early postnatal stages of retinocollicular map refinement. At P0 NrCAM immunostaining was most prominent in the nerve fiber layer (NFL), containing processes of RGC axons, and could also be seen in the developing inner plexiform layer (IPL) (Fig. 1A). Smaller amounts of radially oriented NrCAM staining were evident spanning the retina and ganglion cell layer (GCL) suggested of Mϋller glia labeling. Double immunostaining for L1 showed that NrCAM colocalized in part with L1 in the NFL, and to a lesser extent in the IPL (Fig. 1A). NrCAM was present in the optic nerve head (ON) at lower levels than L1. NrCAM was also localized on fibers of the lens (Fig. 1A), as previously reported [35], and partly colocalized with L1. NrCAM expression in the NFL persisted at P6 but less strongly than L1, but increased in the IPL (Fig. 1A). *In situ* hybridization using anti-sense probes specific for NrCAM and L1 demonstrated the presence of NrCAM and L1 transcripts in cell bodies in the GCL at P0, although it was difficult to assess co-expression even at higher magnification (Fig. 1B). No positive signal was observed in retina with the control sense probe for NrCAM (Fig. 1B). Gradient expression of NrCAM or L1 in the dorsal-ventral retinal axis was not prominent by immunostaining or *in situ* hybridization, though there may be a slight enrichment dorsally (Fig. 1 A,B). Graded expression was not observed in the nasal-temporal axis of the retina (not shown).

In the early postnatal SC (P0), immunostaining for NrCAM and the pan-axonal marker neurofilament NF165 showed NrCAM localized on NF165-positive subpopulations of fibers in the SGS and SO, where RGC axons enter the SC (Fig. 1C). Some NrCAM staining did not colocalize with fibers in the SC. *In situ* hybridization at P0 confirmed that NrCAM was also expressed in cell bodies located in the SC at P0 (Fig. 1D). The expression of NrCAM in early postnatal retina is in accord with its expression in RGCs at embryonic stages [16]. The pattern of expression of NrCAM in the retina and SC during postnatal stages of RGC axon pathfinding in the SC suggested that it may be involved in retinocollicular targeting in a subpopulation of RGC axons.

### NrCAM Null Mutant Mice Display Mediolateral Retinocollicular Mapping Errors

To investigate whether NrCAM has a functional role in development of retinocollicular topography, we mapped the projections of RGC axons to the SC in wild type (WT) and NrCAM null mutant mice by anterograde axon tracing with the lipophilic dye, DiI, when the map resembles its mature form (P8–10). DiI was focally injected into the midpoint of temporal, nasal, dorsal or ventral quadrants of the peripheral retina in live mice at P8, and labeled RGC projections were analyzed 2 days later in the SC. During retinocollicular map formation, RGC axons along the temporal-nasal axis of the retina project to the anterior-posterior axis of the contralateral SC. Temporal retinal injections label RGC terminals in the anterior SC; nasal injections label terminals in the posterior SC [1], [36]–[39]. Conversely, projections of RGC axons along the D-V retinal axis project to the lateral-medial axis of the SC, such that ventral injections label terminals in the medial SC and dorsal injections label terminals in the lateral SC [4], [21], [22], [40].

**Figure 2:**
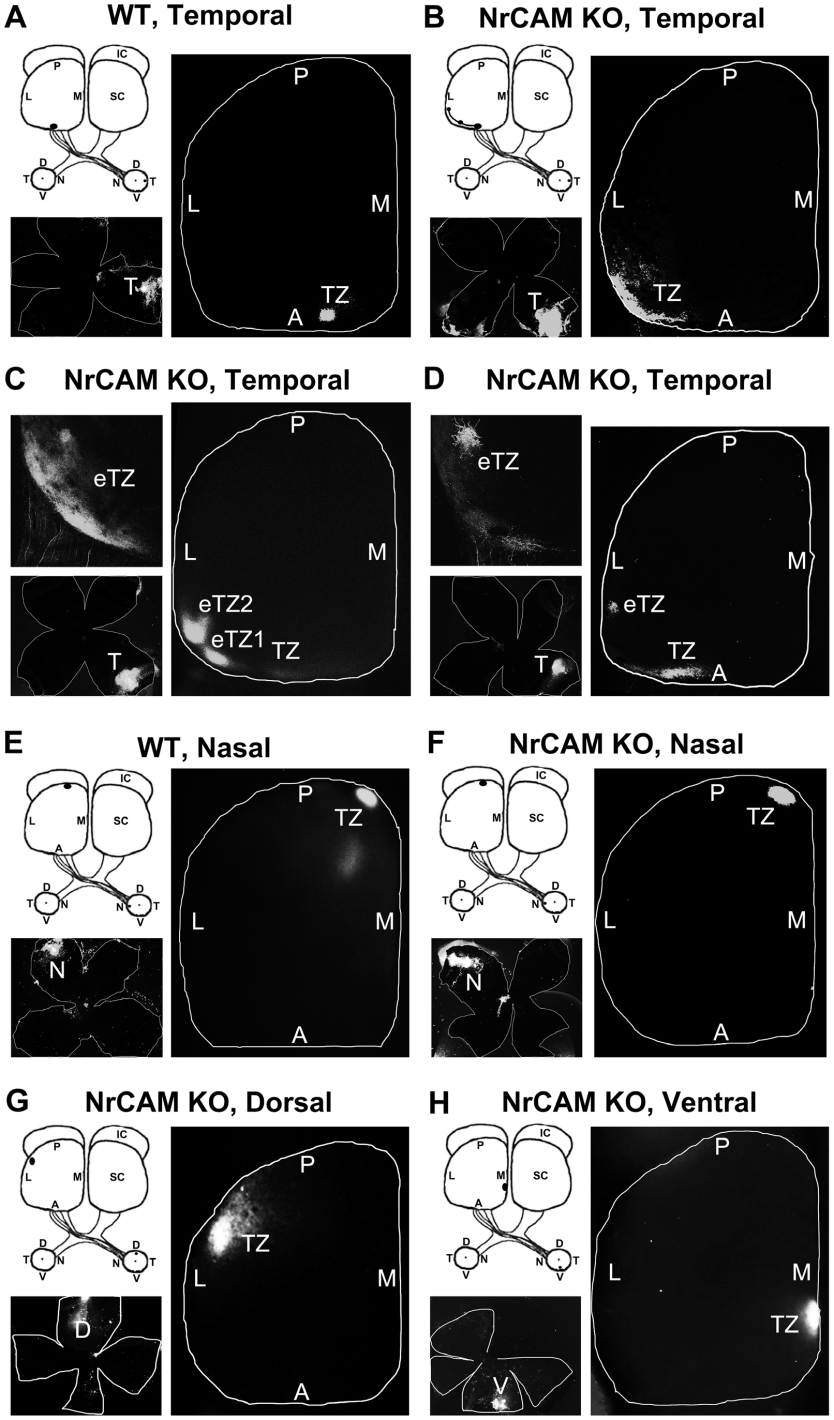
Medial-lateral Targeting Defects of RGC Axons in the Superior Colliculus of NrCAM Null Mice. **A.** In WT mice, DiI injection into the temporal retina at P8 labeled a single dense termination zone (TZ) in the anterior SC at P10. **B–D.** In NrCAM null (KO) mice, DiI injection into the temporal retina labeled multiple laterally displaced ectopic TZs (eTZs) in the SC at P10. **E–F.** DiI injections into the nasal retina labeled a single TZ in the posterior SC that was normally positioned in WT and NrCAM KO mice at P10. **G–H.** DiI injections into the dorsal (G) and ventral (H) retina of NrCAM KO mice resulted in normally positioned single TZs in the lateral (G) and medial (H) SC, respectively. The location of DiI injections were shown in retinal flat mounts in the lower left of each image. L, lateral; M, medial; A, anterior; P, posterior; D, dorsal; V, ventral; T, temporal; N, nasal.

**Figure 3:**
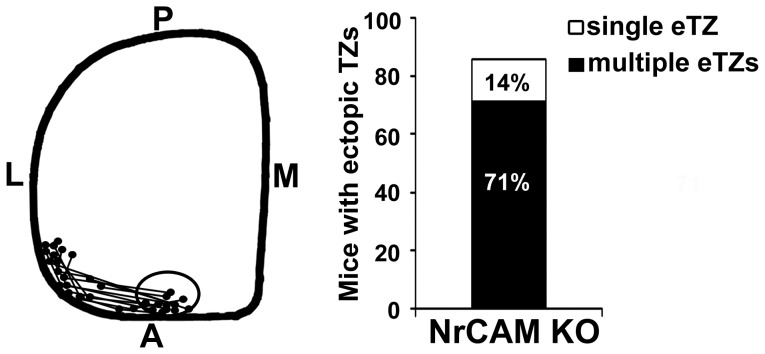
Distribution and Percentage of Termination Zones of Temporal RGC axons in the NrCAM Null Superior Colliculus. **Left panel:** The schematic diagram illustrates the location of TZs of temporal RGC axons in the NrCAM null mutant (KO) SC (P10). The centers of TZs and eTZs from NrCAM KO mice (n = 14) are marked and connected. The distribution of normal TZs of temporal RGC axons in WT mice are all found within the anterior region of the SC depicted by the oval. L, lateral; M, medial; A, anterior; P, posterior. **Right panel:** Percentage of abnormally distributed single and multiple eTZs of temporal RGC axons in the SC of NrCAM null mice (P10). In NrCAM KO mice 85% of mice showed abnormally located eTZs. A single laterally displaced eTZ is found in 14% of mutants, and multiple eTZs were found in 71% of the mutants.

DiI injections into the mid-temporal (T) retina of WT mice labeled a single dense termination zone (TZ) in the mid-anterior region of the contralateral SC (Fig. 2A). In contrast, temporal injections into the retina of NrCAM null mutant mice resulted in aberrant labeling of RGC axons displaced along the mediolateral axis of the contralateral SC. The location and size of DiI injections are shown in retinal flat mounts. A large majority (12/14) of NrCAM mutants (∼85%) showed lateral mistargeting of temporal RGC axons in the SC, resulting in eTZs (Fig. 2B–D). Most of these (10/14) displayed multiple laterally displaced eTZs in addition to one correctly positioned TZ in the mid-anterior SC (Fig. 2C,D), similar to EphB2/B3 double mutants [21] and L1 mutant mice [17], [18]. A minority of NrCAM mutants (∼14%; 2/14) exhibited a single laterally displaced eTZ without a normally positioned TZ (Fig. 2B), which may result from greater malpositioning of axons to form eTZs or to individual variation in NrCAM mutant mice. In addition, the more laterally eTZs were displaced in the SC of NrCAM null mice, the more likely they were to be slightly shifted posteriorly (Fig. 2B–D). A schematic representation of TZs of temporal RGC axons from NrCAM null mutants depicts their relative positions in the SC, compared to the normal distribution of WT TZs (circled area in mid-anterior SC) (Fig. 3).

We also examined retino-collicular targeting of RGC axons from other quadrants of the retina. The targeting of nasal RGC axons to the SC was not perturbed in NrCAM null mice. Nasal injections into the NrCAM mutant retina (7/7) labeled a normally positioned single TZ in the posterior region of the contralateral SC at the same location as in WT mice (Fig. 2E,F). Ventral or dorsal RGC axons of NrCAM mutant mice also terminated at appropriate locations in the SC. Dorsal injections of NrCAM mutant retinas (6/6) resulted in labeling of a single TZ in the lateral SC (Fig. 2G). Similarly, projections from ventral retina of NrCAM null mice (7/7) terminated as a single TZ in the medial SC (Fig. 2H).

Thus, topographic targeting of RGC axons from the temporal retinal quadrant, but not from nasal, dorsal or ventral retina, was sensitive to loss of NrCAM and resulted in single or multiple eTZs that were laterally displaced along the mediolateral axis of the SC.

### Interstitial Branches of Remodeling RGC Axons are Misoriented in the NrCAM Null Superior Colliculus

**Figure 4:**
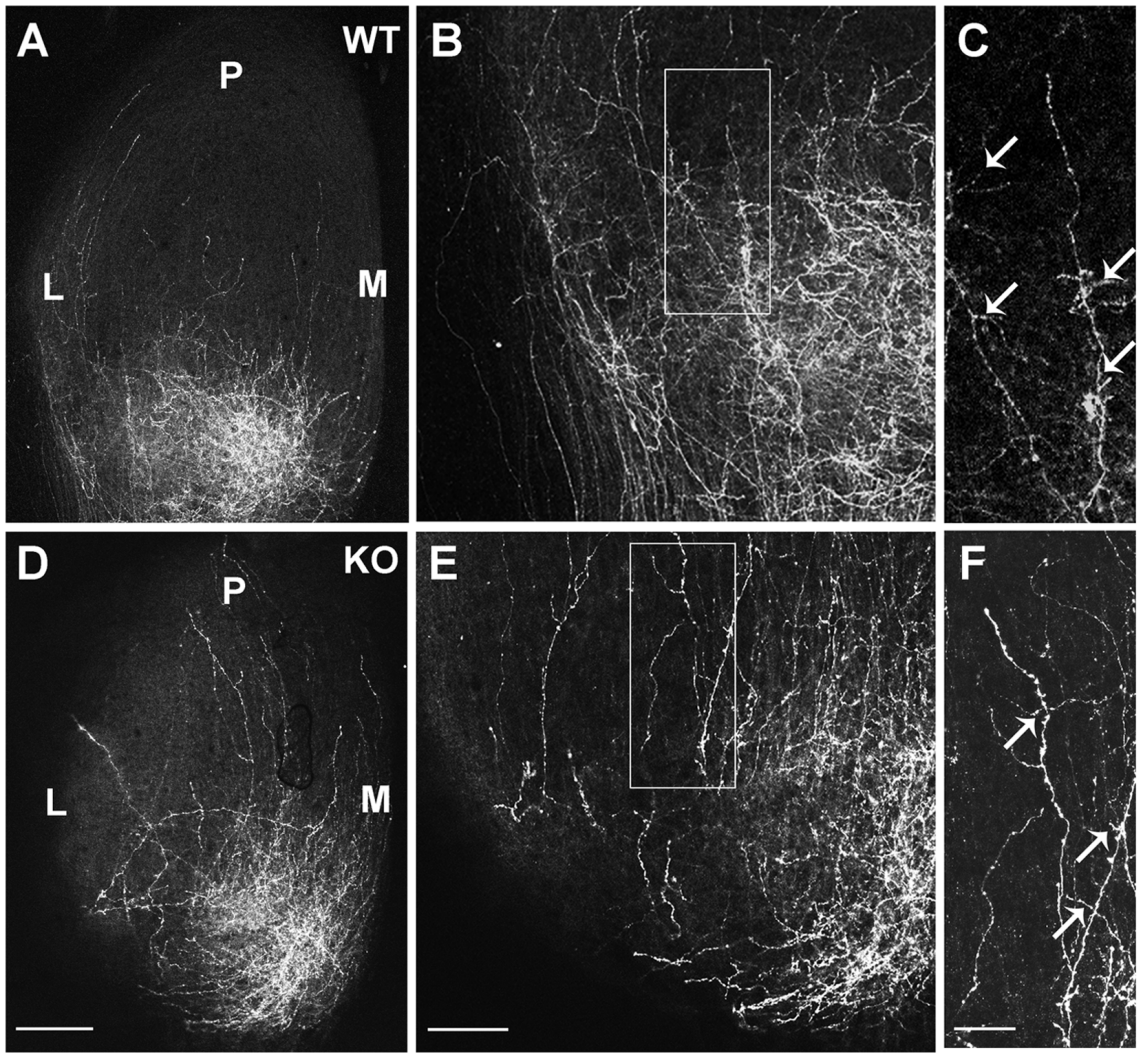
Interstitial Branching of Ventrotemporal RGC axons in WT and NrCAM null SC at P3. **A–C.** DiI labeling of VT RGC axons in WT mice showed that most branches from VT axons in the lateral zone of the SC oriented medially to the future SC, as seen in a higher magnification of the boxed area in B (arrows). **D–F.** DiI labeling of VT axons in NrCAM null mutants (KO) revealed more laterally oriented branches in axons within the lateral zone of the SC, as seen in a higher magnification of the boxed area in E (arrows). M, medial; L, lateral; P, posterior. Scale bar: 200 µm in A,D; 100 µm in B,E; 50 µm in C,F.

During remodeling of overshooting RGC axons in the SC, interstitial branches form along the axon shaft (∼P2) with a mediolateral bias for the topographically correct site of their future TZ by a balancing mechanism between attractant and repellent forces including ephrinB/EphB and Wnt3/Ryk signaling [4], [21], [40]. Because RGC axons from the ventrotemporal (VT) retina form TZs more medially within the anterior SC than mid-temporal axons, any bias for misoriented branches in mutants would be more evident in this subpopulation. To investigate the mechanism for incorrect TZ positioning of RGC axons in NrCAM null mutants, we labeled VT axons of WT and NrCAM null mice with DiI at P2 and quantitatively analyzed interstitial branches in the SC at P3. In WT mice DiI-labeled VT axons were diffusely spread along the mediolateral axis of the SC and extended into the posterior SC (Fig. 4A–C). Interstitial branches from WT axons preferentially oriented toward the correct terminal location in the anteromedial SC, as shown by medially directed branches of axons in the lateral SC (Fig. 4C, arrows). In contrast, interstitial branches from NrCAM null mutant axons tended not to be correctly oriented (Fig. 4D–F), as shown by many laterally oriented branches in the lateral SC (Fig. 4F, arrows).

**Figure 5:**
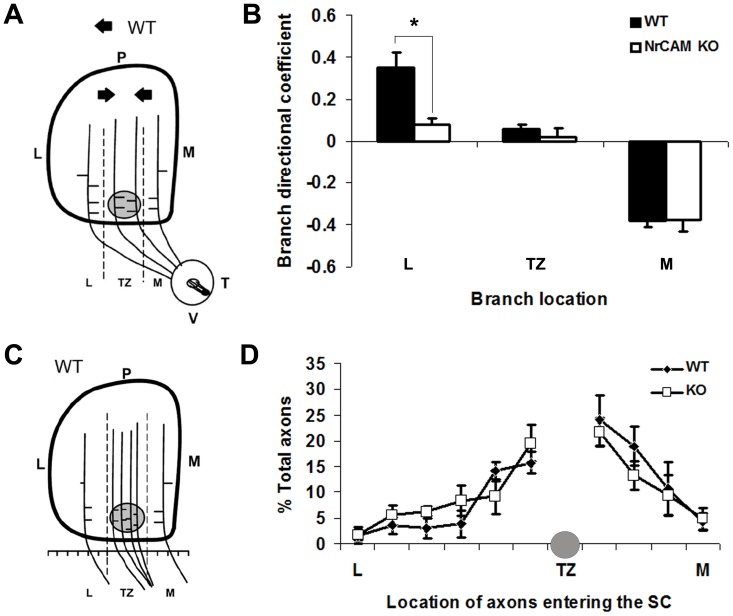
Branching and Entry Position of RGC Axons in the Superior Colliculus of WT and NrCAM Null Mice. **A.** Diagram of the location and branch orientation of DiI-labeled axons from the VT retina within the SC of WT mice at P3. The SC was divided into 3 bins along the medial-lateral axis as shown by the dashed lines: lateral to TZ (L), within TZ (TZ), and medial to TZ (M). The shaded circle represents the site of the forming TZ. Branches were scored and their orientations were analyzed within each bin. The arrows represent the preferred orientation of WT branches. **B.** Branch distribution of VT RGC axons in WT and NrCAM null mutant (KO) mice. The distribution of branches was expressed as a branch directional coefficient for each bin by subtracting the number of laterally oriented branches from medially oriented branches, then dividing by the total number of branches. In WT (n = 4) and NrCAM null (n = 4) mice, 184 and 205 branches were analyzed, respectively. Brackets indicate S.E.M., and asterisks show significant differences (ANOVA, p<0.05). **C.** Diagram of entry position of VT RGC axons in the SC of WT mice at P3. The SC was divided into 10 equal segments along the medial-lateral axis at its anterior border relative to the position of the forming TZ: lateral to TZ (L), within TZ (TZ), and medial to TZ (M). The position of labeled RGC axons in each segment was scored and expressed as the percent of total axons. **D.** Distribution of DiI-labeled VT RGC axons of WT and NrCAM null mutant (KO) mice entering the anterior SC (expressed as the percent of total axons) in bins along the mediolateral axis, as described in **C.** Shaded circle indicates region near the location of normal TZ. In WT (n = 4) and NrCAM null (n = 4) mice, 146 and 167 axons were analyzed, respectively. Error bars represent SEM. There were no significant differences between WT and mutant at any location (ANOVA, p<0.05).

For quantification of branch orientation (Fig. 5A), the SC was divided into three bins along the medial-lateral axis relative to the location of the emerging TZ as follows: lateral to TZ (L), within TZ (TZ), and medial to TZ (M). In each bin, the total number of labeled RGC axons and branches was measured, and medial or lateral branch orientation of each branch was scored (Fig. 5A**–**B). A directional coefficient (DC) was calculated by subtracting the number of laterally oriented branches from medially oriented branches, and dividing the result by the total number of branches, as previously described [18], [19], [21]. A positive DC indicated that there were more medial than lateral branches; a negative DC indicated more lateral than medial branches. This analysis revealed that interstitial branches of VT axons in WT mice (n = 4) preferentially oriented along the mediolateral axis toward the position of the future TZ (Fig. 5A,B), confirming previous studies [18], [19], [21]. Most branches in the lateral bin were oriented medially with respect to the TZ, as indicated by a positive DC; branches within the TZ were largely unbiased; and most branches in the medial bin were laterally oriented to the TZ, as indicated by a negative DC (Fig. 5B). In contrast, many more interstitial branches of VT axons in the lateral bin of NrCAM null mice (n = 5) were oriented laterally, as indicated by a decreased DC compared to WT. (Fig. 5A,B). Branch orientation was significantly different in the lateral bin of WT and NrCAM null mice (ANOVA; P<0.01), but not in the TZ and medial bins (P>0.05). These results suggested that loss of NrCAM resulted in a reduced ability of interstitial branches of VT axons to be attracted medially to the future TZ, a process shown to require ephrinB1/EphB signaling, L1-ankyrin interaction, and ALCAM [18], [19], [21], [41].

To evaluate whether loss of NrCAM resulted in altered patterns of RGC axon entrance into the SC, we measured the distribution of DiI labeled VT axons entering into the SC at P3. For each mouse, the entrance zone of the SC was divided into ten equal bins: four bins located medial and six bins located lateral to the correct TZ (Fig. 5C). The percentage of axons entering the SC in each bin of WT (n = 4 mice; 146 axons) and NrCAM knockout mice (n = 4 mice, 167 axons) was calculated and analyzed by ANOVA. The entry position of VT axons in WT and NrCAM null mice was enriched in the vicinity of the correct TZ and decreased medially as well as laterally. There was no significant difference in the location of WT and NrCAM null mutant axons entering the SC (Fig. 5D). Thus, it is unlikely that laterally shifted TZs in the NrCAM null mice were caused by impaired axonal entrance into the SC.

### EphB2 Phosphorylates NrCAM at the FIGQY Motif and Inhibits Ankyrin Binding

**Figure 6:**
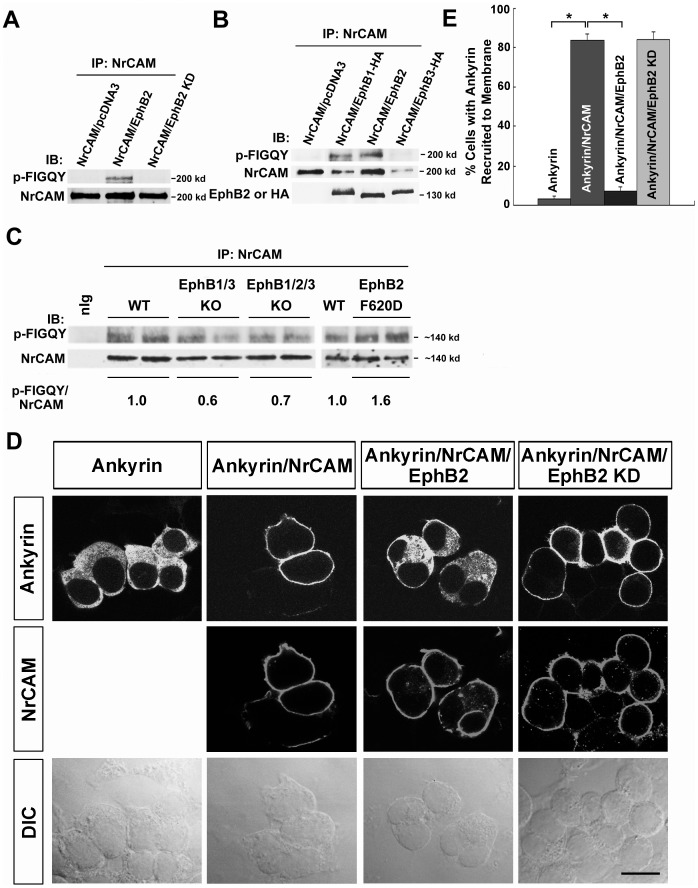
EphB receptors Induce Tyrosine Phosphorylation of NrCAM at the FIGQY Motif, and Inhibit Ankyrin Recruitment in HEK293 cells. **A.** NrCAM was expressed with or without EphB2 or EphB2 kinase dead mutant (EphB2 KD) in transfected HEK293 cells, followed by immunoprecipitation with NrCAM antibodies and immunoblotting with phospho-FIGQY antibodies. EphB2 induced phosphorylation of NrCAM (200 kD) at FIGQY (p-FIGQY) compared to NrCAM alone or to co-expression of the EphB2 kinase dead mutant. Blots were stripped and reprobed with antibodies to NrCAM to indicate relative levels of NrCAM in the immunoprecipitates. **B.** NrCAM was expressed with or without EphB1, EphB2, or EphB3 in transfected HEK293 cells, followed by immunoprecipitation with NrCAM antibodies and immunoblotting with phospho-FIGQY antibodies. EphB1 (HA-tagged) or EphB2 (not HA-tagged) induced effective phosphorylation of NrCAM at FIGQY, whereas EphB3 (HA-tagged) had a weak effect. Blots were stripped and reprobed with antibodies to EphB2 or to the HA-epitope on EphB1 and EphB3. Each EphB receptor was co-immunoprecipitated efficiently with NrCAM. **C.** Lysates of the superior colliculus from WT, EphB1/3 double null, EphB1/2/3 triple null and constitutively active EphB2 (F620D) homozygous mice (P2-P3) were immunoprecipitated with antibodies to NrCAM, and immunoblotted with phospho-FIGQY or NrCAM antibodies. Bands were quantified using the threshold function of ImageJ. The numbers under the blots represent the normalized average phospho-FIGQY/NrCAM band intensity from individual mice. Phosphorylation of NrCAM at FIGQY was reduced in the SC of EphB1/3 and EphB1/2/3 null mice compared to WT, while phospho-FIGQY on NrCAM was increased in the EphB2 F620D mice. D. Cytofluorescence assay for recruitment of ankyrinG-EGFP to NrCAM in the plasma membrane of transfected HEK293 cells was visualized by confocal imaging. Boxes above images indicate the expression plasmids used for transfection, while boxes at the left indicate fluorescence labeling of ankyrinG-EGFP or NrCAM in the same cells. Corresponding differential contrast (DIC) images are shown below. AnkyrinG-EGFP localized within the cytoplasm of cells expressing ankyrin alone, while co-expression of NrCAM led to the recruitment of ankyrinG-EGFP to the cell membrane, where NrCAM was localized. When NrCAM was co-expressed with EphB2, ankyrinG-EGFP remained largely present in the cytoplasm. When NrCAM was co-expressed with EphB2 KD, ankyrinG-EGFP was recruited to the plasma membrane where NrCAM was localized in most cells, although occasionally some cytoplasmic localization was seen. Scale bar = 15 µm. E. Quantification of the percentage of cells with ankyrinG-EGFP recruited to the plasma membrane of HEK293 cells transfected as in **C**. NrCAM expression significantly increased ankyrinG-EGFP recruitment to the plasma membrane in ankyrin/NrCAM-expressing cells as compared with ankyrin expression alone (Ankyrin/NrCAM: 84±3%; Ankyrin: 3±1.6%). In cases of ankyrin/NrCAM/EphB2 co-expression, ankyrinG-EGFP recruitment was strongly decreased (7±2%) compared to ankyrin/NrCAM expression. There was no significance decrease in ankyrinG-EGFP recruitment in cases of ankyrin/NrCAM/EphB2 KD co-expression (84±4%). Error bars show S.E.M. Asterisks indicate significant differences in means (one-way ANOVA, Tukey’s post-hoc test, *p<0.001).

Laterally displacement of TZs of temporal RGC axons in the NrCAM null SC resembled the mediolateral retinotopic phenotype of EphB mutant mice [21], [22]. EphB2 is the most prominent EphB receptor exhibiting dorsal-low to ventral-high gradient in the mouse retina [21], [22]. The tyrosine residue within the L1 FIGQY motif is phosphorylated by EphB receptor kinases, decreasing L1-ankyrin association [41]. However, there are substantial differences in the cytoplasmic domains of NrCAM and L1, including the presence of a carboxyl terminal PDZ binding domain in NrCAM [42], [43]. Therefore, we investigated whether EphB receptor tyrosine kinases were able to phosphorylate NrCAM at the cytoplasmic sequence. NrCAM and EphB2 expression plasmids were co-transfected into HEK293 cells for transient expression, and NrCAM was analyzed for tyrosine phosphorylation within the FIGQY sequence by immunoprecitation of NrCAM followed by immunoblotting with a phospho-specific p-FIGQY antibody, which was previously characterized [41]. Results demonstrated that EphB2 effectively induced tyrosine phosphorylation of NrCAM at the FIGQY motif, while there was no endogenous phosphorylation of NrCAM in cells that were transfected with NrCAM plasmid alone (Fig. 6A–B). In addition, an EphB2 kinase dead mutant (EphB2K662R; EphB2 KD) [24] did not mediate phosphorylation of NrCAM at the FIGQY motif (Fig. 6A), indicating that the kinase activity of EphB2 was essential for FIGQY phosphorylation. Similar transient expression of EphB1 (hemagglutinin (HA)-tagged) or EphB3 (HA-tagged) showed that EphB1 was also effective at inducing tyrosine phosphorylation of NrCAM at FIGQY, whereas EphB3 was much less effective (Fig. 6B). Interestingly, each EphB receptor (B1, B2, B3) co-immunoprecipitated with NrCAM from transfected cells (Fig. 6B), demonstrating a molecular association between NrCAM and EphB receptors.

To evaluate the consequence of altered EphB expression on NrCAM phosphorylation at FIGQY *in vivo*, SC lysates were prepared from WT, EphB1/3 double null, EphB1/2/3 triple null and homozygous mice (P2**–**P3) expressing constitutively active EphB2 (F620D). NrCAM was immunoprecipitated from the lysates, immunoblotted with phospho-FIGQY antibodies, then reprobed with NrCAM antibodies. Phosphorylation of NrCAM at FIGQY was reduced in the SC of EphB1/3 and EphB1/2/3 null mice compared to WT, while phospho-FIGQY on NrCAM was increased in the EphB2 (F620D) mice with overactive kinase activity (Fig. 6C). These results showed that NrCAM can be phosphorylated on FIGQY in the SC during topographic mapping dependent on EphB receptors. The residual phosphorylation in EphB1/3 and EphB1/2/3 triple mutants may be due to other Eph receptor kinases, or there may be compensatory phosphorylation by EphB receptors when one is deleted.

To evaluate the ability of EphB2 to regulate NrCAM-ankyrin binding, we used a cytofluorescence assay that measures recruitment of EGFP-labeled ankyrinG (ankyrinG-EGFP) from the cytoplasm to NrCAM in the cell membrane of transfected HEK293 cells [29]. Like L1, NrCAM is a transmembrane cell adhesion protein which localizes to the HEK293 cell membrane, as indicated by red fluorescence (Fig. 6D, second row). Expression of ankyrinG-EGFP alone resulted in a diffuse distribution of EGFP fluorescence in the cytoplasm, whereas co-transfection of NrCAM with EGFP-ankyrinG led to recruitment of EGFP fluorescence to the cell membrane where NrCAM was localized (Fig. 6D). When EphB2 was co-transfected with NrCAM and ankyrinG-EGFP under conditions shown to induce tyrosine phosphorylation of the NrCAM FIGQY motif, ankyrinG-EGFP remained cytoplasmic (Fig. 6D). In contrast, co-expression of the EphB2 KD mutant with NrCAM and ankyrinG-EGFP, which does not induce NrCAM phosphorylation at FIGQY, resulted in the recruitment of ankyrinG to the cell membrane (Fig. 6D). Quantification of percentage of cells with ankyrin recruited to the cell membrane showed that greater than 80% of cells with NrCAM/ankyrinG co-expression displayed membrane recruitment of ankyrinG, and that this was significantly greater than in cells with ankyrinG expression alone (3%) (Fig. 6E). In EphB2/NrCAM/ankyrinG transfections, the percent of cells exhibiting ankyrinG recruitment to the cell membrane was significantly decreased, compared with NrCAM/ankyrinG transfections, and not different from ankyrinG transfection alone (Fig. 6E). In cells expressing EphB2 KD with NrCAM/ankyrinG, the percent of cells with ankyrinG recruitment to cell membrane was equivalent to cells with NrCAM/ankyrinG expression (Fig. 6E). These results demonstrate that phosphorylation of NrCAM at the FIGQY motif by EphB2 kinase is associated with inhibition of ankyrin recruitment to the plasma membrane, where NrCAM is localized.

In summary, EphB2 induced tyrosine phosphorylation of NrCAM at the ankyrin binding domain (FIGQY) and inhibited ankyrin binding, consistent with an ability of EphB2 to modulate the association of NrCAM with the actin cytoskeleton. Such modulation may contribute to RGC interstitial branch orientation in the SC in response to a high medial to low lateral ephrinB2 gradient, which facilitates topographic targeting of a subpopulation of RGC axons along the mediolateral axis of the SC.

## Discussion

Here we show that NrCAM contributes to retinocollicular topography by regulating mediolateral targeting of temporal RGC axons in the contralateral SC. Loss of NrCAM resulted in pronounced lateral displacement of TZs of these axons within the SC, and impaired interstitial branch orientation during refinement of projections in development. EphB receptors, which regulate mediolateral retinocollicular axon targeting, associated with and phosphorylated NrCAM on tyrosine within the ankyrin binding motif (FIGQY), decreasing ankyrin binding in an *in vitro* cell model. These results suggest a molecular mechanism in which reversible linkage of NrCAM to the actin cytoskeleton through EphB phosphorylation promotes attraction of RGC axon branches toward a medial-high ephrinB gradient in the SC, ensuring appropriate mediolateral synaptic targeting of RGC axon subpopulations.

The mediolateral targeting defects in NrCAM null mice closely resembled those of mouse mutants in EphB1–3 and ephrinB1–2, as well as L1 and ALCAM. Each of these mutants display laterally displaced TZs of temporal and ventrotemporal RGC axons [18], [19], [21], [22]. EphB2/EphB3 mutant mice [21], L1 [17], [18], and ALCAM [19] null mice also exhibit impaired medial orientation of interstitial axon branches, similar to NrCAM mutants. EphB receptors associated with and differentially phosphorylated NrCAM (EphB1, EphB2> EphB3) on the regulatory tyrosine residue of the FIGQY ankyrin binding motif, decreasing ankyrin recruitment in a kinase-dependent manner, as shown in transfected HEK293 cells. *In vivo*, the high penetrance of mapping errors made by temporal axons in the NrCAM null SC approximated that reported for EphB1/EphB2 and EphB2/EphB3 double mutants, but was greater than EphB2 or EphB3 single mutants [21], [22], or EphB2 (F620D) homozygotes [22]. Accordingly, levels of NrCAM phospho-FIGQY were decreased in the early postnatal SC of EphB1/B3 double mutants and EphB1/B2/B3 triple mutants, and increased in EphB2 (F620D) homozygotes. These findings suggest that NrCAM is likely an effector of ephrinB signaling from multiple EphB receptors important for topographic targeting of RGC axons.

L1 is also phosphorylated by EphB receptors at its FIGQY ankyrin binding domain, disrupting ankyrin recruitment [41]. However, EphB receptors differentially phosphorylated NrCAM and L1. EphB1 and EphB2, which have large effects on temporal axon mapping [22], phosphorylated both NrCAM and L1 [41], whereas EphB3, which has a smaller effect on mapping [22], phosphorylated L1 [41] and to a much lesser extent NrCAM. It is therefore possible that different combinations of EphB receptors expressed on temporal RGC axon subpopulations modulate NrCAM- and L1-dependent adhesion to different degrees to achieve refined regulation of retinocollicular targeting. The role of NrCAM in retinal axon growth and branch orientation is in accord with its ability to induce growth cone protrusions in developing chick RGCs [44]. The expression of NrCAM on retinal axons as well as on cells in the retino-recipient layers of the SC suggests that homophilic interactions may also contribute to targeting.

EphrinB2 and EphB1 promote repulsion of early-stage (E14) VT retinal axons at the optic chiasm to form the ipsilateral projection independent of NrCAM [45]–[47]. However, NrCAM is required for a late-crossing subpopulation of VT axons to cross the optic chiasm, as shown by aberrant ipsilateral projection in NrCAM null mice [16]. The temporal axons mapped in our study were not derived from this population, as they terminated in the contralateral SC. NrCAM did not appear to influence retinal axon outgrowth *per se,* because RGC axons grew to the SC, which they reached at an appropriate age. Furthermore, NrCAM also did not alter targeting or segregation of ipsilateral or contralateral projections to the lateral geniculate nucleus of the dorsal thalamus [15]. Normal targeting of ventral and dorsal RGC axons, as well as axon entry into the SC, in NrCAM null mice indicated that pre-target axon sorting of these axons, which chiefly controls mediolateral mapping of ventral RGC axons [48], was most likely unaffected. Similarly, EphB2/EphB3 null [48], ALCAM null [19], L1 [17], and L1Y^1229^H mutant mice [18] show normal pre-target axon sorting.

Countergradients of Neuropilin-2 on RGCs and Semaphorin3F in the SC are suggestive of a potential role in anterior-posterior retinocollicular mapping [49]. NrCAM associates with the Semaphorin3F receptor Neuropilin-2 to mediate repulsive guidance of thalamocortical [15] and anterior commissural axons [50]. A small shift in anterior-posterior location of eTZs in the NrCAM null SC suggested that NrCAM/Neuropilin-2 interactions might play a minor role in anterior-posterior mapping, or alternatively reflect competitive interactions between normal and laterally displaced axons [51]. Because loss of NrCAM did not alter the lateral orientation of interstitial branches, it is unlikely to be involved in the repellent counterforce mediated by Wnt3/Ryk signaling [4].

The present research supports a mechanism in which NrCAM regulates mediolateral retinocollicular mapping of temporal axon subpopulations in response to ephrinB-EphB signaling to promote directional branch extension vital to correct synaptic targeting and development of an appropriate retinotopic map. Phosphorylation of NrCAM at the FIGQY motif by EphBs in RGC axons may decrease ankyrin binding and cytoskeletal linkage, reducing their affinity for substrate-bound ligands such as ALCAM, thereby promoting branch attraction toward the medial high ephrinB gradient in the SC.

The widespread expression of NrCAM and EphBs along axonal projections in the brain suggests a potentially general role for the mechanism described here in establishment of synaptic connectivity and topography in specific regions of the developing nervous system.
